# A Thermally Annealed Mach-Zehnder Interferometer for High Temperature Measurement

**DOI:** 10.3390/s140814210

**Published:** 2014-08-04

**Authors:** Zhongyao Feng, Jiacheng Li, Xueguang Qiao, Ling Li, Hangzhou Yang, Manli Hu

**Affiliations:** Physics Department, Northwest University, No.229, Taibai Road (North), Xi'an 710069, China; E-Mails: jcli@stumail.nwu.edu.cn (J.L.); xgqiao@nwu.edu.cn (X.Q.); lli@stumail.nwu.edu.cn (L.L.); yanghz@nwu.edu.cn (H.Y.); huml@nwu.edu.cn (M.H.)

**Keywords:** fiber-optic sensor, Mach-Zehnder interferometer (MZI), high temperature

## Abstract

An in-fiber Mach-Zehnder interferometer (MZI) for high temperature measurement is proposed and experimentally demonstrated. The device is constructed of a piece of thin-core fiber (TCF) sandwiched between two short sections of multimode fiber (MMF), *i.e.*, a MMF-TCF-MMF structure. A well-defined interference spectrum is obtained owing to the core-mismatch, and the interference dips are sensitive to the ambient temperature. The experimental results show that the proposed interferometer is capable of high temperature measurement up to 875 °C with a sensitivity of 92 pm/°C over repeated measurements. The explored wavelength drop point may limit the measurement range, which can be improved by repeated thermal annealing.

## 1. Introduction

Fiber-optics high temperature sensors have attracted great interest for monitoring the temperature in extreme environments such as turbines, combustors, nuclear reactors and aerospace systems. To date, diverse interferometers have been widely proposed for their excellent high temperature resistance or high sensitivity. Fiber-optic Fabry-Perot interferometers (FFPIs), as interference devices, generally show positive features such as high stability, tiny size and simple fabrication, which have attracted significant attention in high temperature sensing [1–3]. Taking a case, an in-line FFPI, in which a short section of polarization-maintaining photonic crystal fiber (PM-PCF) is spliced with an end-cleaved single-mode fiber, has been employed to measure the temperature up to 600 °C with a sensitivity of 13.8 pm/°C [1]. However, FFPIs generally present low temperature sensitivity, which will limit the measurement resolution. In comparison, some fiber interferometers based on modal interference can effectively improve the temperature sensitivity in the high temperature range. A sandwiched fiber structure, *i.e.*, a thin-core fiber, spliced between two single mode fibers (SMFs), has been proposed to measure high temperatures up to 850 °C [4]. Besides, the in-line Mach-Zehnder interferometer (MZI) as another smart interferometer based on modal interference also presents excellent high temperature features. Multiple MZIs have been developed to measure high temperatures, such as using a piece of multimode fiber (MMF) sandwiched between a single mode fiber (SMF) and a sensing tapered SMF [5], and inserting a micro-hole into the fiber using a femtosecond laser [6], *etc.* Although these devices can withstand high temperatures, they are costly and complex to fabricate. Some core-mismatch structures can outperform them in the fabrication aspect (showing the compact size and simple fabrication), for example, a thin-core followed by a multimode fiber structure has been developed to measure temperature [7]. According to the description above, some MZIs can be employed to measure high temperature, however, they often suffer from dopant diffusion or frozen-in stress release inside the fiber and this disturbs the stability and repeatability of the sensing devices. This may be an intrinsic characteristic of this kind of MZI, to which attention should be paid and the devices improved. A sapphire fiber-based interferometer that is free from dopant diffusion has been demonstrated for high temperature measurement up to 1600 °C [8]. The annealing process does not significantly affect the temperature performance. In our prior work, we have proposed a reflective probe using a polarization-maintaining photonics crystal fiber (PMF-PCF) [9]. Although the PMF-PCF formed by pure silica is also free from the dopant diffusion issue, the high temperature can release the frozen-in stress inside the fiber and eventually results in hysteresis during the heating and cooling processes. After several annealing operations, the PMF-PCF-based interferometer can measure temperatures up to 1100 °C and present good repeatability. Some fiber Bragg grating (FBG)-based devices can measure temperatures up to 1000 °C with good stability and repeatability. Their fabrication may include tailoring the glass composition [10], specific thermal or annealing processes on conventional gratings [11,12] and inscribing gratings using femtosecond lasers [13]. However, their low temperature sensitivities limit their practical applications. Another type of technique is based on long period gratings (LPGs), which present smart transmission spectra because of the cladding-mode-based coupling mechanism [14]. Besides, cascading two or more LPGs can result in interference spectra based on multiple mode interference [15,16]. These cascaded configurations can be used to measure multiple parameters simultaneously since the interference dips present different sensitivities to different surrounding perturbations such as refractive index, strain and temperature. Although, the LPGs-based devices present outstanding performance, they cannot tolerate the high temperature, which limits their applications in harsh high temperature environments [15,17].

In this paper, we propose and experimentally demonstrate a compact in-fiber MZI for high temperature measurement made by a simple fabrication process by splicing a TCF between two short sections of MMFs. The high-order cladding modes can be excited via the core-mismatch of MMF-to-TCF, and then the cladding and core modes are coupled into the downstream SMF by the second MMF. A clear superimposed interference pattern is obtained as the result of cladding and core mode interference. Several thermal annealing processes are carried on the MZI to release the frozen-in stress, which significantly improves the thermal reliability and stability. After thermal processing, the MZI interference dip shows a good linear response to the temperature change with a sensitivity of 92 pm/°C, which is higher than that of a similar structure reported previously owing to the large MMF-to-TCF core-mismatch. In conclusion, we also present a feasible way to not only extend the sensing range of the structure, but also to confirm its repeatability and reliability, makes it a good candidate for high temperature measurement.

## 2. Operation Principle

**Figure 1.**
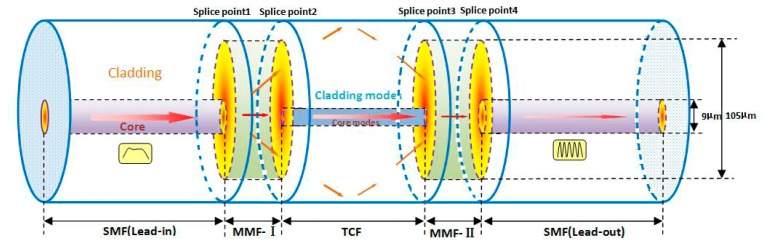
Schematic diagram of the MZI structure.

Figure 1 demonstrates the schematic diagram of the proposed MMF-TCF-MMF structure. An uncoated TCF is spliced between two short pieces of MMF. The MMFs are similar to the mode couplers with the core/cladding diameter of 105 μm/125 μm, which are used to split and recombine the light signal due to the core-diameter mismatch. The TCF works as the interference arm with core/cladding diameter of 5.5 μm/120 μm. In detail, the first MMF couples part of the core-guided fundamental mode into the cladding of the downstream TCF via the mismatched fiber core cross-section. The coupled cladding modes pass through the TCF sandwiched with a controlled length. Finally, the cladding modes are coupled back to the fiber core of lead-out SMF via the second MMF, mixing with the original core mode and interfere with the guided core mode.

According to the above description, the interference intensity can be given as:

(1) $$I=I_{\text{core}}+\sum_{m}I_{\text{cladding}}^{m}+\sum_{m}2\cdot \sqrt{I_{\text{core}}\cdot I_{\text{cladding}}^{m}}\cdot \cos\Phi^{m}$$

where *I*, *I_core_*, $I_{\text{cladding}}^{m}$ are the intensities of interference light, the light propagating in the fiber core and *m^th^* cladding mode and Φ*_m_* is the phase delay. The interference pattern depends on the phase delay resulting from light transmitting along the cladding and core of TCF with different refractive indices. Of which, the phase difference between the core and cladding modes after propagating through TCF can be written as:

(2) $$\Phi^{m}=\frac{2\pi (n_{\text{eff}}^{co}-n_{\text{eff}}^{cl,m})L}{\lambda }=\frac{2\pi \Delta n_{\text{eff}}^{m}L}{\lambda }$$

where $n_{\text{eff}}^{co}$, $n_{\text{eff}}^{cl,m}$ are the effective refractive indices of core and *m^th^* cladding mode, *_L_* is the length of TCF, λ is the signal wavelength in vacuum, and $\Delta n_{\text{eff}}^{m}$ is the effective index difference, respectively. According to the interference principle, the corresponding the interference valley wavelength should be calculated as:

(3) $$\lambda_{m}=\frac{2\Delta n_{\text{eff}}^{m}L}{\left(2m+1\right)}$$

where *m* is an integer. According to the Equation (3), the wavelength separation Δλ between two interference dips can be approximated as:

(4) $$\Delta \lambda =\frac{4\Delta n_{\text{eff}}^{m}L}{\left(2m+1\right)\left(2m-1\right)}\approx \frac{\lambda^{2}}{\Delta n_{\text{eff}}^{m}L}$$

According to Equation (4), the free spectral range (FSR) decreases with increasing TCF length.

**Figure 2.**
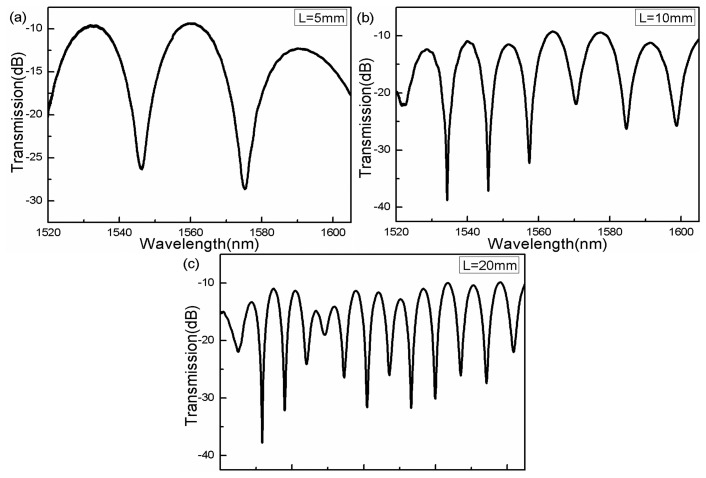
Transmission spectra of MMF-TCF-MMF configuration with different TCF lengths.

Figure 2 shows the interference spectra of the interferometers with the 5 mm, 10 mm, and 20 mm-long TCFs. The length of MMFs are fixed at L_0_ = 3 mm. Since the phase deviation is very small around the phase-matching wavelength, the dip transmission of the interferometer can be expressed as cos^2^*kL*_0_ according to the coupled mode theoretical analysis, where k is the coupling coefficient between core mode and cladding mode and L_0_ is the length of MMF. Therefore, the different lengths of the MMFs could influence excitement of cladding modes and the transverse field distribution at the splice interface, and thus determine the coupling strength of the core-to-cladding modes.

By trial and error, the 3 mm-long MMFs contribute to the coupling of core-to-cladding modes and, eventually we obtain a well-defined interference spectrum with a high fringe contrast and low intensity loss. The inhomogeneous interference pattern verifies that the interference spectra result from more than two modes participating in the interference. The exciting cladding modes are highly dependent on the splice points between MMFs and TCF. The splice point 2 dominates the transverse field distribution at the splice interface and thus determines the coupling strength of upstream MMF core mode to the TCF core/cladding modes and splice point 3 contributes to couple the modes to form the interference pattern.

**Figure 3.**
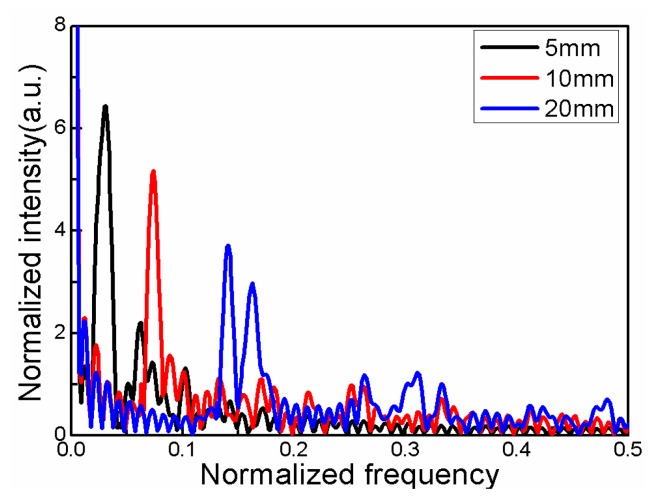
Spatial spectra of several interference spectra in Figure 2.

In order to further analyze the characteristics of the interference pattern, the interference pattern is transformed into the corresponding spatial Fourier spectrum. As shown in Figure 3, many cladding modes are involved in the interference. The spatial frequency is shown as:

(5) $$\xi =\frac{1}{\lambda^{2}}\Delta m_{\text{eff}}L$$

where *ξ* is the spatial frequency, λ is the center wavelength, Δ*m_eff_* is the differential modal group index and *L* is the length of TCF. According to the Equation (5), *ξ* is proportional to a group index difference and the length of TCF. In Figure 3, three main intensity peaks indicate that there is one dominant cladding mode and other weakly excited ones. To the case of *L* = 5 mm, the spatial frequency of the mainly excited cladding mode is located at *ξ* =0.03124, the other excited ones are weak, such as the location around *ξ* =0.11123. To our knowledge the strongly excited cladding mode is a low-order mode and the weak excited ones are in high-order. The high-order cladding modes modify the main interference pattern as shown in Figure 3.

With a changing ambient temperature, the mode group indices of the core and cladding modes change differently due to the different thermo-optic dependence of the fiber core and cladding. This will contribute to the phase delay difference, and eventually result in wavelength shift. According to Equation (3), the wavelength shift can be written as:

(6) $$\delta \lambda_{m}=\frac{2L}{\left(2m+1\right)}\delta \Delta n_{\text{eff}}^{m}+\frac{2\Delta n_{\text{eff}}^{m}}{\left(2m+1\right)}\delta L$$

Equation (6) shows that δλ*_m_* is proportional to the TCF length variation δ*L* and the group mode effective index difference variation $\delta \Delta n_{\text{eff}}^{m}$.

## 3. Experimental Results and Discussion

**Figure 4.**
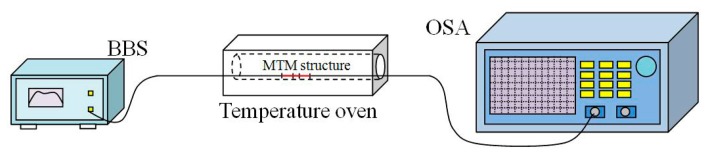
Schematic diagram of the experimental setup for SRI measurement.

Figure 4 shows the schematic diagram of the temperature sensing system. A MTM structure is put into a temperature oven. A MTM with the length of TCF *L* =5 mm is fabricated and employed to measure the temperature, which the interference spectrum presents large free spectrum range (FSR). During the temperature operations, the interference spectrum shows red-shift with temperature increase and blue-shift with temperature decrease.

**Figure 5.**
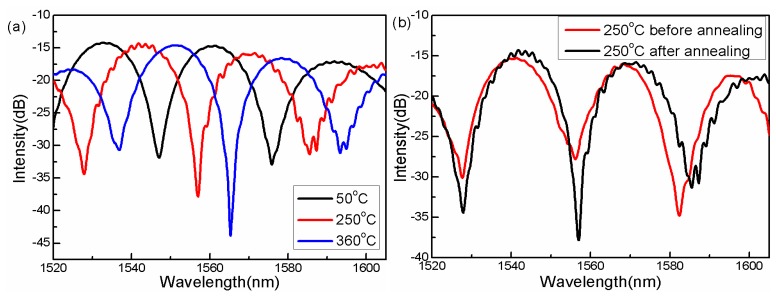
(**a**) Interference spectrum dip responses to the increasing temperature; (**b**) interference spectra before and after annealing processes.

An interference dip is employed to monitor the temperature change in the oven, while each temperature point is held for a certain time to ensure the temperature stability in the oven before the data is recorded. During the first heating process, some ripples appeared on the smooth interference spectrum at 250 °C, and then disappeared at a temperature of 360 °C, as shown in Figure 5a. After the annealing operation, the interference spectrum can effectively smoothed, *i.e.*, the spectral ripples do mostly disappear after annealing, as seen by comparing with the spectrum before annealing operation, as shown in Figure 5b. The ripples on the spectrum are a consequence of the high-order cladding modes participating in the interference, which are excited at the fusion interface due to the relaxation of the stress during the first heating process. When the stress is gradually released and new equilibrium is formed, the ripples disappear, as theoretically expected.

**Figure 6.**
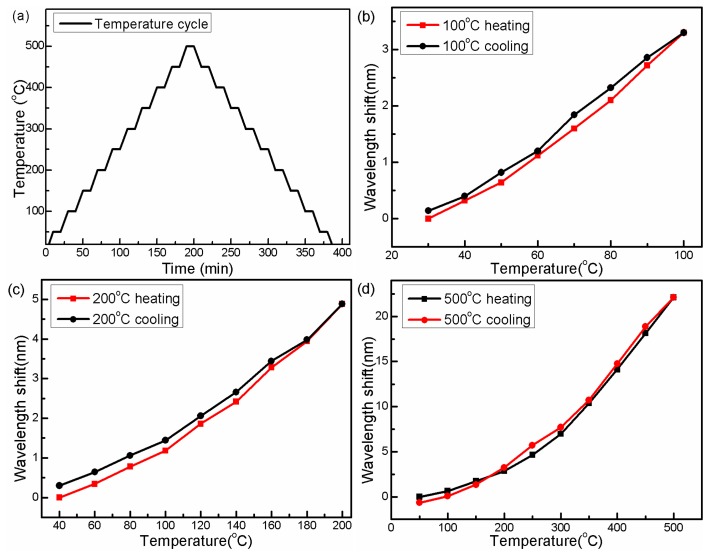
(**a**) Temperature cycle operation. Interference dip wavelength *versus* the temperature during the different cycles with the maximum temperature of (**b**) 100 °C, (**c**) 200 °C and (**d**) 500 °C.

In order to further release the stress, several annealing processes have been implemented with maximum temperatures of 100 °C, 200 °C and 500 °C. All the annealing processes are performed at a rate of 5 °C/min and each temperature point is held for 10 min as shown in Figure 6a–c show that the interferometer presents a certain hysteresis during the heating and cooling processes in the lower temperature range from 30 °C to 100 °C and 40 °C to 200 °C. In Figure 6d, with the annealing temperature increasing to 500 °C, this small thermal hysteresis is canceled out, and the temperature response of the proposed device shows good repeatability.

**Figure 7.**
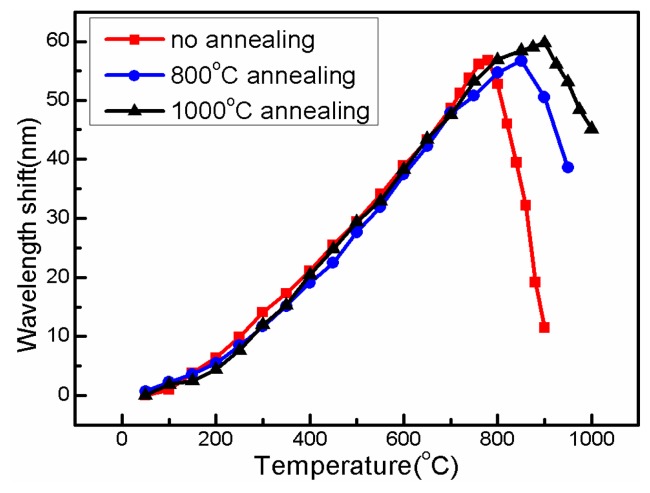
Measured interference dip wavelength *versus* temperature under different annealing conditions: no annealing (**Red line**, blue-shift point at 780 °C), 800 °C annealing (**Blue line**, blue-shift point at 850 °C), 1000 °C annealing (**Black line**, blue-shift point at 900 °C).

When the temperature is higher than 780 °C, the interference dip rapidly shifts to a shorter wavelength, as shown by the red line in Figure 7. This is due to the relaxation of the internal stresses “frozen” in the fiber during drawing [18]. This sharp “blue-shift” limits the temperature measurement range and repeatability of the proposed sensor. In order to improve the sensing capacity, several annealing cycles with maximum temperatures of 600 °C, 700 °C and 800 °C, respectively, are employed to thermally deal with the sensing device. After these thermal annealing operations, the “blue-shift point” has been successfully pushed up to 900 °C, as the black line shown in Figure 7. It is also seen that the dip wavelength is a linear function of temperature at the range of 25 °C to 800 °C, and the corresponding temperature sensitivity is 92 pm/°C.

**Figure 8.**
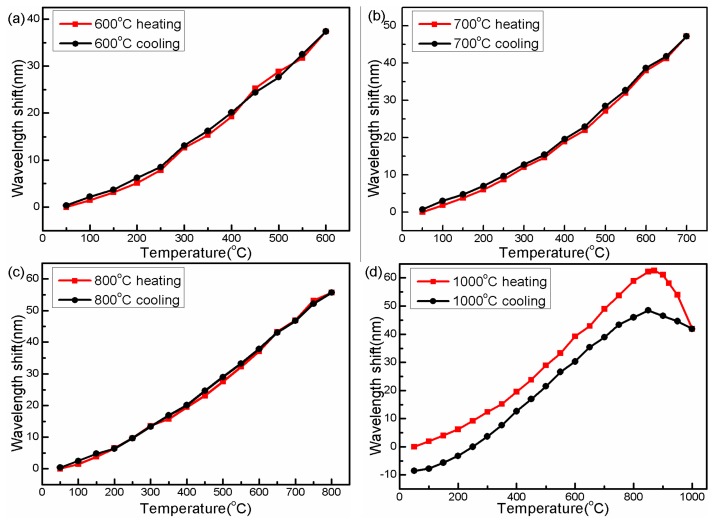
Interference dip wavelength *versus* the temperature during the different cycles with the maximum temperature of (**a**) 600 °C, (**b**) 700 °C, (**c**) 800 °C, and (**d**) 1000 °C.

The wavelength “blue-shift point” also worsened the temperature measurement repeatability of the sensing device. As shown in Figure 8a–c, temperature curves during the heating and cooling processes show perfect repeatability below 800 °C. However, when the annealing temperature is raised to 900 °C, a clear gap occurs between the heating and cooling curves, as shown in Figure 8d. This unrepeatability problem (caused by the thermal hysteresis effect) limits the reliability of the proposed sensor. In order to eliminate the drawback, the following steps of high temperature annealing operations are carried out.

**Figure 9.**
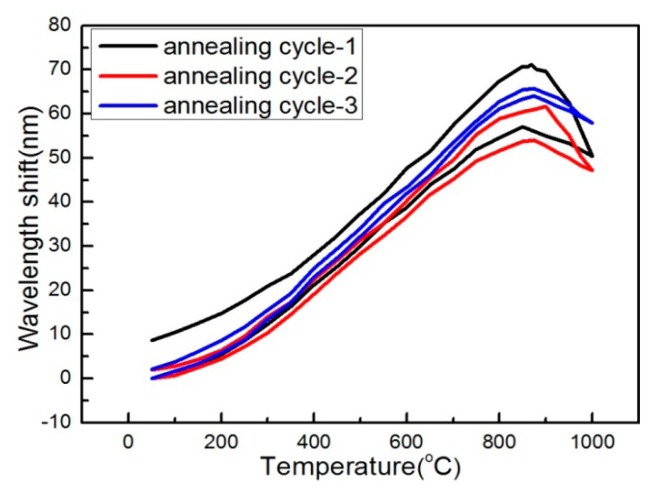
Wavelength responses of MZI sensor under several heating and cool temperature cycles (from 25 °C to 1000 °C).

The plots of interference dip wavelengths *versus* several temperature cycles at high temperatures ranging from 50 °C to 1000 °C are shown in Figure 9. A clear separation exists between the heating and cooling wavelength response curves, as the black line (annealing cycle-1) shows in Figure 9. For normal cylindrical fibers, the thermal hysteresis is directly proportional to the glass internal stresses. To further reduce the influence of thermal hysteresis, repeated annealing operations are employed. As shown in Figure 9, after annealing cycles-1 to 3, the thermal hysteresis has been significantly decreased, and the thermal repeatability has also been improved.

**Figure 10.**
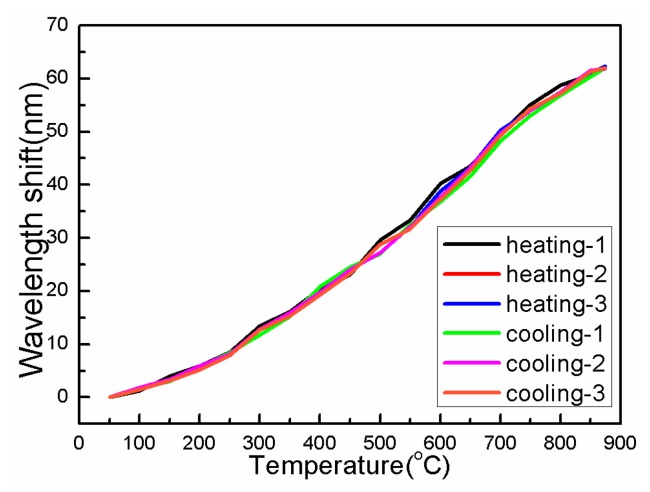
Cumulative wavelength responses of MZI sensor during heating and cooling processes from 25 °C to 875 °C.

After a series of annealing operations, three temperature operations of heating and cooling from 25 °C to 875 °C are carried out to further verify the repeatability of the proposed MTM sensor, in which each temperature operation is employed for an interval of 72 h (three days). Figure 10 shows the experimental result whereby the MTM sensor now presents a significant repeatability during the heating and cooling processes.

Based on the above experimental results, the following conclusions can be made: when the annealing temperature is below 780 °C, the dip wavelength response to temperature shows perfect stability, linearity and repeatability. With rising temperature, the wavelength “blue-shift” phenomenon occurs due to the “frozen” stress release in silica. When the “frozen” stress releases instantaneously, the refractive index of the proposed configuration will sharply change [19], which can affect the phase difference and cause the observed “blue-shift”. With continuous release of the “frozen” stress by annealing, the “blue-shift point” is pushed to higher temperatures. However, once the annealing temperature rises to the glass transmission region, the nonequilibrium glass structure will gradually transmit to the equilibrium liquid. The physical state change speeds up the internal stress release and changes the refractive index of the proposed structure as well. With the implementation of several annealing operations, the “frozen” stress in the fiber will be further released to the limitation of the fiber melting point. The “blue-shift point” dominated by physical state transmission is finally fixed at a temperature of 875 °C. After these annealing processes, the influence of the internal stress inside the fiber can be eliminated well, and the measurement range, stability and repeatability of the sensing device can be improved.

## 4. Conclusions

We propose a simple in-fiber MMF-TCF-MMF MZI sensing configuration for high temperature measurement. Its interference spectrum is employed to monitor the ambient temperature change. An annealing process is used to improve the measurement range, stability and repeatability of the MZI device. After several annealing operations, the sensor presents a linear temperature sensitivity of 92 pm/°C over a large temperature range from 25 °C to 875 °C with good stability and repeatability. The proposed sensor has the advantages of compact size, simple structure, high stability and low cost, making it a good candidate for small and distributed sensing in high temperature sensing applications.
